# En Route Toward Sustainable Polycarbonates via Large Cyclic Carbonates

**DOI:** 10.1002/cssc.202500030

**Published:** 2025-06-09

**Authors:** Francesco Della Monica, Arianna Brandolese, Graziano Di Carmine, Maurizio Selva, Giulia Fiorani, Lorella Izzo

**Affiliations:** ^1^ Department of Biotechnology and Life Science University of Insubria Via Jean Henry Dunant, 3 21100 Varese Italy; ^2^ School of Chemistry University of Birmingham Birmingham B15 2TT UK; ^3^ Department of Environmental and Prevention Science University of Ferrara 44121 Ferrara Italy; ^4^ Department of Molecular Sciences and Nanosystems Ca’ Foscari University of Venice Via Torino, 155 30172 Venezia Italy

**Keywords:** catalysis, circularity, cyclic carbonates, diols, polycarbonates

## Abstract

This review reports an up‐to‐date overview of the synthetic methodologies developed for the preparation of large cyclic organic carbonates with ≥6‐membered rings (6M‐CCs and above), highlighting the most sustainable synthetic pathways employing diols (including renewable‐based ones) and nonhazardous carbonyl sources, e.g., linear organic carbonates, in mild operating conditions. The lower thermodynamic stability of 6M‐CCs compared to 5‐membered ones allows for a straightforward preparation of biocompatible aliphatic polycarbonates (APCs), occurring via ring‐opening polymerization (ROP), in the presence of various active organo‐ and/or biocatalysts. Moreover, ROP processes can be tuned for the selective preparation of copolymers with different thermomechanical properties and can be further applied to structurally complex, larger cyclic carbonate derivatives. Finally, the end‐of‐life fate of APCs, particularly the recently reported controlled depolymerization strategies, is critically discussed focusing on chemoselectivity toward cyclic carbonate or epoxide monomers. This timely overview highlights the open challenges as well as the opportunities associated with the synthesis of APCs and chemical recycling and highlights their potential as circular and sustainable plastics.

## Graphical abstract

The use of safe carbonylating agents, allowed by a suitable (bio)catalytic systems, affords the sustainable and green synthesis of large‐ring cyclic carbonates by reaction of renewables such as bio‐based diols. These carbonates are prone to ring‐opening polymerization paving the way for the production of circular aliphatic polycarbonates.

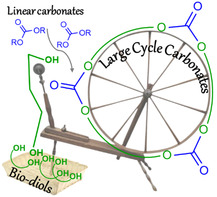

## 1 Introduction: Limitation of CO_2_/Epoxides Insertion Reactions

Chemical valorization of carbon dioxide (CO_2_) is a timely and challenging research topic, closely linked to the development of renewable‐based value chains and climate change mitigation strategies. The past few years have witnessed the rise of the importance of CO_2_ as C1 building block, exploiting its reactivity with strained heterocycles, including epoxides, oxetanes, aziridines, and episulfides.^[^ ^1^, ^2^ ^]^ Epoxides represent a relatively large family of commercially available, structurally diverse scaffolds which can be synthesized from either fossil‐ and renewable‐based feedstock, and in some specific cases are established industrially relevant feedstocks (i.e., ethylene oxide, EO, and propylene oxide, PO).^[^ ^3^ ^]^

**Scheme 1:**
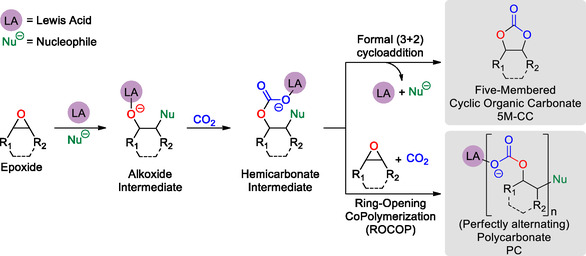
Divergent reactivity pathways for CO_2_/epoxide coupling reactions.

The main pathway toward the formation of 5‐membered cyclic organic carbonate (5M‐CC) and polycarbonates (PCs) from epoxides and CO_2_ is presented in **Scheme** **1**.^[^ ^4^ ^]^ At first, a Lewis acidic catalyst (LA) activates the epoxide and a nucleophile opens the epoxy ring yielding an alkoxide intermediate. After, CO_2_ insertion into a LA‐alkoxide bond leads to the formation of a hemicarbonate intermediate. At last, the hemicarbonate can either form the corresponding 5M‐CC, obtained upon formal (3 + 2) cycloaddition, or PC, obtained upon consecutive epoxide/CO_2_ enchainment via ring‐opening copolymerization (ROCOP).

The reaction of CO_2_ with epoxides can be considered an established synthetic route to selectively obtain CCs and PCs. In the past few years, the synthesis of both CCs and PCs via ROCOP of epoxides and CO_2_ has reached a high level of sophistication, as witnessed by the number of publications in the field and as summarized in some excellent review articles on CO_2_ valorization applications.^[^ ^2^, ^4^, ^5^, ^6^, ^7^, ^8^, ^9^, ^10^ ^]^ Moreover, CO_2_/epoxide coupling reactions encompass many desirable sustainable features: 1) both CC synthesis and ROCOP occur with a 100% atom economy; 2) the reactions are thermodynamically favorable, however, to overcome CO_2_ kinetic inertness, active catalytic systems must be designed and employed (vide infra); 3) reactions are mostly carried out in solventless conditions; 4) epoxides coreagents can be fully derived from the renewables pool, as will be discussed in detail for the production of poly(limonene carbonate) (PLC); and 5) upon optimization of the reaction conditions and/or of the catalytic system, CO_2_ insertion occurs with a high degree of chemoselectivity toward the formation of the CC or PC products.

Interestingly, both CCs and PCs allow long‐term storage of CO_2_ in useful products, such as solvents and functional materials, in contrast to short‐lived CO_2_ storage, which is currently occurring for key CO_2_‐derived industrial products, such as urea. CCs are a class of functional compounds characterized by tuneable reactivity and by a range of physicochemical properties compatible with their application as solvents.^[^ ^11^, ^12^, ^13^ ^]^ Industrially relevant CCs include: ethylene carbonate (EC), which is currently employed in the Asahi Kasei industrial process as a greener alternative for the synthesis of bisphenol A (BPA)‐based PCs through a transcarbonation reaction between EC and methanol to produce dimethyl carbonate (DMC);^[^ ^14^, ^15^ ^]^ propylene carbonate (*c*PC) mainly employed as solvent due to its wide liquid temperature range and suitable as solvent component for lithium‐ion batteries; glycerol carbonate which can be fully sourced from renewable‐based starting materials and contains both polar aprotic and polar protic groups within its structure, suitable for applications as solvent.^[^ ^12^, ^16^ ^]^

Intriguingly, CCs can react chemoselectively in the presence of nucleophilic coreactants: in agreement with Pearson's hard–soft acid–base theory, “hard” amines and diamines nucleophiles undergo preferentially carbonyl attack to form the corresponding urethanes, while “soft” nucleophiles like thiols favor alkylation.^[^ ^17^, ^18^ ^]^ More recently, the reactivity of structurally complex CCs was studied in the presence of Pd catalysts and in conditions favoring rearrangement reactions.^[^ ^19^ ^]^

PCs are divided into two groups: aromatic PCs and aliphatic PCs (APCs). Aromatic PCs are mainly represented by BPA‐based PCs where carbonate linkages are bound to bulky aromatic phenyl rings bridged by quaternary carbon atoms. BPA‐based PCs are engineered thermoplastics characterized by physicochemical properties suitable for practical applications, such as high glass transition temperatures (*T*_g_) (*T*_g_ = 150–170 °C), high strength at low temperatures, flame retardancy, thermal stability, and UV–vis transparency. BPA‐based PCs present several commercial applications including data storage, electronics, optical components, construction materials, and automotive industry.^[^ ^20^, ^21^ ^]^ Conversely, APCs have been less commercialized and are not used as thermoplastics, but are typically included as comonomers and/or oligomeric intermediates for the synthesis of polyurethanes or other copolymers. This is ascribed to the poorer thermomechanical properties of APCs compared to aromatic PCs, largely influenced by the type of flexible aliphatic linkage connecting the repeating carbonate units, i.e., low rigidity in the case of poly(propylene carbonate) (PPC), and brittleness in the case of poly(cyclohexene carbonate) (PCHC) (vide infra).^[^ ^22^, ^23^ ^]^ Nevertheless, in recent years APCs have been studied as biocompatible materials for drug delivery, polymer‐based therapeutics, and imaging contrast agents, as they are more biocompatible and (bio)degradable compared to BPA‐PCs and can be obtained from a wide range of natural and renewable‐based sources. Moreover, APCs are characterized by versatile chemical structures, and they can undergo selective postpolymerization functionalization.^[^ ^24^ ^]^

Open challenges in PCs chemistry are the replacement of BPA as comonomer and the development of phosgene‐free polymerization routes.^[^ ^23^ ^]^ Interestingly, the range of APCs which can be obtained by epoxide/CO_2_ ROCOP is quite diverse and includes (bi)cyclic scaffolds, e.g., cyclohexene oxide (CHO), limonene oxide (LO), and linear monomers such as EO and PO. A strong limiting factor associated with the synthesis of PCs via the ROCOP of CO_2_ and heterocyclic substrates is that the resulting copolymers are characterized by C_2_ linkers between carbonate units for CO_2_/epoxides ROCOP, or by C_3_ linkers in the case of the much less studied CO_2_/oxetanes ROCOP.

The selective PCs versus CCs formations can be controlled with catalytic system design and/or choice of heterocyclic monomer. For example, chemoselective synthesis of PPC from PO and CO_2_ is controlled by the type of catalytic system employed: PPC is thermodynamically less stable than polypropylene glycols that can be readily prepared homopolymerizing PO and less stable than the cyclic *c*PC product. Therefore, designing an active and chemoselective catalytic system for the synthesis of PPC requires: 1) maximizing the yield of PC product with minimal formation of polyether and *c*PC by‐products; 2) minimal polymer purification procedure to obtain transparent and colorless PPC; 3) control over molecular weight (*M*_n_), polydispersity (*Đ*), and polymer microstructure; and 4) the catalytic system should be nontoxic.^[^ ^22^ ^]^

Although the quest for such catalytic systems is still ongoing, there are a few relevant milestones in the design of catalytic systems for selective PPC synthesis, promising also for large‐scale applications, as PO is currently used to make polyether polyols and manufactured on a 12 Mt year^−1^ scale. Noteworthy, replacing polyether polyols with PPC would drastically reduce the emission of polymer production, as PPC contains up to 43 wt% of CO_2_.^[^ ^25^ ^]^ Besides the pioneering studies reported by Coates and co‐workers on PO/CO_2_ ROCOP catalyzed by Co(III)salen(Cl)/PPNCl catalytic system,^[^ ^26^ ^]^ requiring the presence of cocatalysts, multinuclear macrocyclic catalysts are probably the most active and selective catalytic systems for PPC production.^[^ ^27^, ^28^, ^29^ ^]^ The main contribution in this area is from Williams and co‐workers, who developed a library of Co(III)‐based heterodinuclear macrocyclic catalysts. Interestingly, the Co(III)/K(I) macrocyclic complex is one of the most active and selective complexes for PO/CO_2_ ROCOP, displaying activity at 0.001 mol% catalyst loading under mild experimental conditions (*T* = 50 °C, *p* ^0^(CO_2_) = 5.0 bar, *t* = 84 min, PPC yield = 96%, carbonate linkages >99%).^[^ ^25^ ^]^ A combined computational and experimental investigation highlighted the distinct role of each metal embedded in the heterobimetallic macrocyclic catalyst toward PPC selectivity: while Co(III) activates PO and stabilizes the alkoxide and hemicarbonate catalytic intermediates, K(I) forms a transient carbonate nucleophile that ring‐opens the activated PO.^[^ ^30^ ^]^

It was observed that for the coupling of CO_2_ and terminal epoxides, CCs are the thermodynamic products, due to the high activation energy barrier for CC ring‐closure, requiring long reaction times, reaction *T* ≥ 80 °C and nucleophile availability, while PCs are the kinetic products, requiring high turnover frequency (TOF) catalysts and mild reaction conditions. Interestingly for coupling reactions involving CO_2_ and di‐ or trisubstituted epoxides, the observed reactivity patterns are somewhat different, especially for alicyclic epoxides. Taking CHO as a model for disubstituted alicyclic epoxides, it was observed by Darensbourg and others that the activation barrier for cyclohexene carbonate (CHC) synthesis is significantly higher (≈80 kJ mol^−1^) compared to that of CHO/CO_2_ ROCOP: this is ascribed to the ring strain placed on the 5M‐CC product to accommodate the conformational requirements of the alicyclic cyclohexyl ring.^[^ ^5^, ^31^, ^32^ ^]^

PLC is probably the most well‐known example of renewable‐based PC, obtained upon ROCOP of CO_2_ and LO, a trisubstituted alicyclic renewable‐based epoxide. LO can be obtained upon chemoselective oxidation of naturally occurring (*R*)‐limonene, a monoterpene which is the main component of citrus peel oil, a by‐product of citrus fruit processing. Limonene contains two endocyclic stereocenters; therefore, upon oxidation, two different LO diastereoisomers are obtained, *cis*‐ and *trans*‐(*R*)‐LO.

Back in 2004, Coates and co‐workers reported the first example of LO/CO_2_ ROCOP, obtained in the presence of a *μ*‐bis(iminato) zinc acetate complex as the catalyst.^[^ ^33^ ^]^ Stereo‐ and regioregular alternating PLC was obtained from a *cis*/*trans* LO mixture (*M*_w_ = 10.8·10^3^ g mol^−1^, *Đ* = 1.12, [cat] = 0.4 mol%, *T* = 25 °C, *p* ^0^(CO_2_) = 0.6 MPa, *t* = 4 h, TOF = 37 h^−1^): remarkably, *trans*‐LO was copolymerized preferentially (% *trans* in copolymer = 98.9%). High molecular weight PLC samples were produced with longer reaction times and higher [epoxide]/[Zn] ratios. Although this copolymerization system is active and selective, the stability of the Zn‐bis(imidate) catalyst is limited, as the catalyst is easily deactivated by protic impurities, e.g., diol by‐products and/or adventitious water. In an effort to improve catalyst stability and scale up PLC synthesis, Greiner and co‐workers developed an optimized ROCOP protocol, increasing the molecular weight of PLC to <10^5^ g mol^−1^ by synthetically masking hydroxyl impurities in the *trans*‐LO monomer.^[^ ^34^ ^]^

More recently, Kleij and co‐workers reported on the synthesis of PLC promoted by an Al(III)‐amino(trisphenolate) comple in combination with a nucleophilic halide cocatalyst.^[^ ^35^ ^]^ ROCOP of commercial (*R*)‐LO and CO_2_ was achieved under relatively mild conditions (*T* = 42 °C, *p* ^0^(CO_2_) = 5–10 bar), with PLC containing significant amounts of *cis* units (up to 33%), suggesting that the Al(III)‐amino(trisphenolate) catalyst could copolymerize both LO stereoisomers. Control experiments revealed that copolymerization of pure *cis*‐(*R*)‐LO was faster compared to copolymerization of pure *trans*‐(*R*)‐LO. Nevertheless, *trans*‐(*R*)‐LO enchainment occurred with a higher degree of stereoselectivity, leading to up to 98% *trans* units in the PLC. The latter result was ascribed to the different regioselectivity of the halide‐mediated ring‐opening of *cis*‐(*R*)‐LO, occurring on the more hindered position of the oxirane unit. This hypothesis was supported by detailed computational investigations, which indeed showed that nucleophilic attack onto the α‐position (at the tertiary carbon center) is energetically favored for both LO stereoisomers, on the basis of an electronic bias.^[^ ^35^, ^36^ ^]^ A comparison of the thermochemical properties of PPC, PCHC and PLC is reported in **Table** **1**.

**Table 1:** Comparison of the thermal properties of the main APCs obtained by CO_2_/epoxide ROCOP.

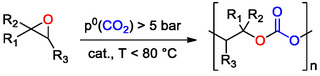		
PO	CHO	*Trans*‐LO
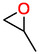	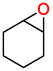	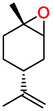
Terminal epoxide Elastomeric PPC	Alicyclic‐disubstituted epoxide Thermoplastic PCHC	Alicyclic‐disubstituted epoxide Thermoplastic PLC
*T*_g_ = 25−45 °C (40 °C)^[^ ^107^ ^]^	*T*_g_ = 125 °C^[^ ^108^, ^109^ ^]^	*T*_g_ = 128 °C^[^ ^110^ ^]^
*T*_d_(onset) = 150−180 °C^[^ ^107^ ^]^	*T*_d_(50%) = 312 °C^[^ ^109^ ^]^	*T*_d_(5%) = 250 °C^[^ ^110^ ^]^

From the critical discussion of the most prominent examples of commercially promising APCs obtained via CO_2_/epoxide ROCOP, it can be noticed that this polymerization technique has some established advantages including the large variety of suitable epoxide scaffolds,^[^ ^3^ ^]^ the possibility of designing ad hoc metal‐based catalytic systems, maximizing chemoselective carbonate enchainment, combining high catalytic activity in mild operating conditions, and producing APCs with high CO_2_ content (up to 43% for PPC). Moreover, the latest findings on catalytic systems active at *p* ^0^(CO_2_) <10 bar suggest the possibility of refitting existing industrial equipment for commercial production of PPC via PO/CO_2_ ROCOP.^[^ ^25^ ^]^

Nevertheless, there are still some relevant limitations which must be overcome for commercial applications of CO_2_/epoxide ROCOP. Among them, the synthetic procedures for epoxides preparation are still characterized by limited scalability, especially for renewable‐based epoxides such as LO, and an extensive purification of the epoxide comonomer is required to minimize the presence of protic impurities which can interfere with the polymerization process by acting as chain transfer agents. Currently, ROCOP catalysts are not commercially available and must be prepared ad hoc and carefully stored, as most of them are sensitive to air and protic impurities, making the synthetic process on a gram scale challenging. From a practical point of view, setting up a ROCOP reaction is quite complicated as in most cases it requires dedicated high‐pressure equipment to handle CO_2_ pressures ≥10 bar. Finally, as reported in Table 1, the thermochemical properties and, hence, the processability windows of APCs obtained via ROCOP are still not optimal for commercial applications, although promising approaches for PCs thermal properties modulation have been recently reported, such as the preparation of terpolymers and/or block copolymers.^[^ ^37^, ^38^, ^39^ ^]^

By contrast, synthesis of APCs with ≥C_3_ linkers can be readily achieved via ring‐opening polymerization (ROP) of the corresponding cyclic carbonates. ROP polymerization can be carried out in living or immortal conditions, depending on the choice of polymerization catalyst and experimental conditions, achieving polymers with controlled and easily predictable *M*_w_ and narrow distributions. Moreover, the experimental setup for ROP polymerization is much easier compared to ROCOP, allowing for the development of metal‐free protocols.^[^ ^40^ ^]^ The main limitation of this approach is the scarce availability of large cyclic carbonates due to their challenging synthesis, ascribed to their limited thermodynamic stability (vide infra).^[^ ^22^ ^]^

**Scheme 2:**
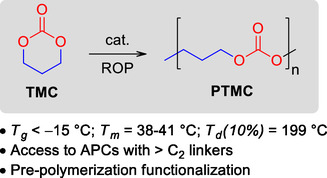
Synthesis of PTMC via ROP of TMC and relevant thermochemical properties.^[^ ^106^ ^]^

Nevertheless, poly(trimethylene carbonate) (PTMC) is a commercially available APC obtained by ROP of the corresponding 6‐membered CC (6M‐CC), trimethylene carbonate (TMC). Although the thermochemical properties of PMTC are typical of a soft and rubbery material (**Scheme** **2**), they are strongly dependent on PTMC *M*_w_, improving sharply for *M*_w_ > 1.0 × 10^5^ g mol^−1^.^[^ ^41^ ^]^

Interestingly, PTMC is biodegradable in vivo by enzymes.^[^ ^42^ ^]^ Therefore, PMTC is mainly employed in biomedical applications since the degradation products are alcohols and CO_2_, preventing potential inflammatory responses.^[^ ^22^ ^]^

## 2 Five‐Membered and Six‐Membered CCs

**Scheme 3:**
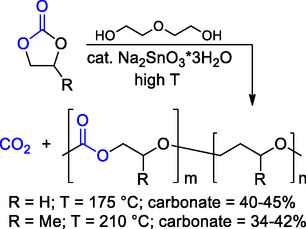
Ethylene and propylene carbonate polymerization.

The ROP of 5M‐CCs is usually very difficult to achieve due to insufficient ring strain, and the prediction of monomer reactivity is not simple.^[^ ^43^ ^]^ For example, EC^[^ ^44^ ^]^ and *c*PC^[^ ^45^ ^]^ usually release carbon dioxide under polymerization conditions, yielding polymers with a low content of carbonate units and resulting in a very difficult control of polymer composition and molecular weights (**Scheme** **3**).

**Scheme 4:**
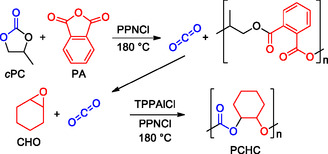
Tandem ROCOP to yield polyester and polycarbonate.

Recently, a tandem polymerization process was proposed coupling PO and CO_2_, released from *c*PC decarboxylation, into two different ROCOP reactions with phthalic anhydride and CHO, respectively (**Scheme** **4**).^[^ ^46^ ^]^ This elegant approach allows the atom economic utilization of *c*PC as monomer, but proves the intrinsic difficulty of modulating the resulting polycarbonate structure when starting from CC monomers. Differently, fused bicyclic 5M‐CCs with sufficient ring strain can lead to the formation of polycarbonates via ROP. However, also in this case structural diversity may lead to different reactivity.

**Scheme 5:**
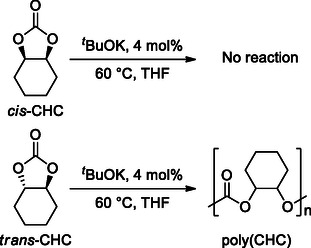
Different reactivity of *cis*‐ and *trans*‐cyclohexene carbonate.

For example, it is known that *cis*‐CHC is inert toward polymerization, while anionic ROP of *trans*‐CHC is possible without decarboxylation (**Scheme** **5**).^[^ ^47^, ^48^ ^]^

On the contrary, ROP of 6M‐CCs such as TMC is very well established. To date, several 6M‐CCs have been employed for the synthesis of APCs via both metal‐ and organocatalysis and this topic has been extensively reviewed.^[^ ^24^, ^40^ ^]^ However, their synthesis is usually based on the use of phosgene or phosgene‐related carbonylation reagents, such as bis(trichloromethyl) carbonate (triphosgene) or bis(pentafluorophenyl)carbonate.^[^ ^49^ ^]^ For example, recently Fukushima reported the synthesis of functional cyclic carbonates from 2,2‐bis(methylol)propionic acid (bis‐MPA), and subsequent ROP toward ether‐functionalized APCs (Scheme 17, vide infra).^[^ ^50^ ^]^ Consequently, the development of synthetic procedures not involving the use of these reactants is highly desirable.

**Scheme 6:**
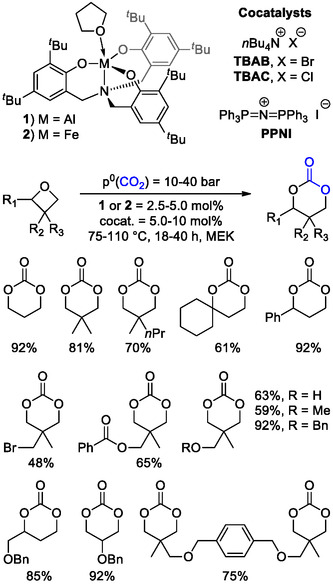
Structures of Al‐ (**1**) and Fe‐ (**2**) aminotriphenolate complexes, and cocatalysts (top) used for the synthesis of substituted 6M‐CCs by reaction of CO_2_ and oxetanes (bottom). Isolated yields are reported for each product.

In principle, 6M‐CCs can be obtained by direct cycloaddition of CO_2_ to oxetanes, in a reaction similar to that of epoxides. However, due to their lower ring strain, this pathway is usually hampered by the low reactivity of oxetanes and a limited number of catalytic systems have been reported to promote this reaction.^[^ ^51^, ^52^, ^53^, ^54^, ^55^ ^]^ Among others, Kleij reported the selective formation of 6M‐CCs from variously substituted oxetanes under relatively mild conditions (*T* = 75 °C, *p*°(CO_2_) = 10 bar) using aluminum (**1**) and iron (**2**) aminotriphenolate complexes in the presence of a nucleophilic cocatalyst such as tetrabutylamminium bromide (TBAB), chloride (TBAC), or bis(triphenylphosphine)iminium iodide (PPNI) (**Scheme** **6**).^[^ ^56^ ^]^

Notably, Buckley et al. reported the electrochemical synthesis of TMC under 1 bar of CO_2_ pressure.^[^ ^57^ ^]^ In this case, high yield and selectivity (96% and 96%, respectively) have been achieved in acetonitrile solution using a single compartment cell with Cu cathode, Mg anode, tetrabutylammonium iodide (TBAI) as supporting electrolyte, and 90 mA current.

The use of oxetanes as the starting material suffers for the intrinsic limitation of narrow structural variety, and different approaches have been proposed to overcome this issue.

**Scheme 7:**
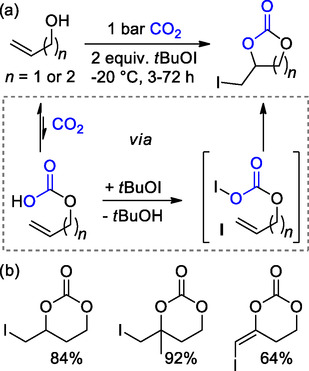
a) CCs formation from unsaturated alcohols and b) 6M‐CCs scope. Isolated yields are presented for each product.

In 2010, Minikata et al. demonstrated the possible fixation of carbon dioxide into iodomethyl‐substituted cyclic carbonates by its reaction with unsaturated alcohols in the presence of *tert*‐butyl hypoiodide (*t*BuOI).^[^ ^58^ ^]^ This method is based on the initial formation of an alkylcarbonic acid from carbon dioxide and the alcohol, followed by the reaction with *t*BuOI yielding an active species **I**; subsequent cyclization of **I** yields the desired CCs (**Scheme** **7**). This procedure allows for CO_2_ use under low‐pressure conditions but is limited to allylic and homoallylic alcohols.

**Scheme 8:**
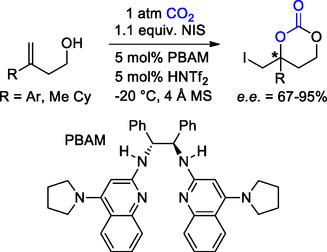
Enantioselective synthesis of iodomethyl‐substituted 6M‐CC.

Notably, Johnston et al. succeeded in the enantioselective synthesis of analogous iodomethyl‐substituted 6M‐CCs via a tandem alcohol carboxylation−alkene iodocarbonation reaction promoted by a Brønsted acid/base combination (**Scheme** **8**).^[^ ^59^ ^]^ Under optimized conditions, CO_2_ reacts with substituted homoallylic alcohols and *N*‐iodosuccinimide (NIS) in the presence of chiral pyrrolidine‐substituted bis(amidine) base (PBAM) and bis(trifluoromethanesulfonyl)amine (HNTf_2_) as the catalyst. More than 15 examples were reported, with yields ranging from 26 to 96% and with moderate to high enantiomeric excess (e.e. = 67–95%).

**Scheme 9:**
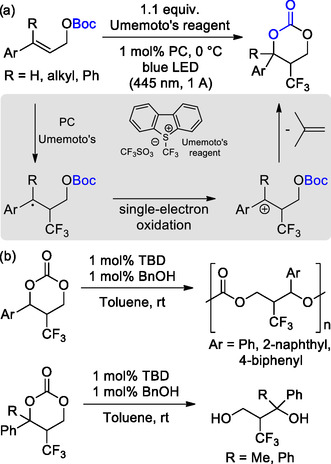
a) Photocatalytic synthesis of 6M‐CCs from γ‐substituted allyl carbonates and b) their organocatalytic ROP.

More recently, Limburg and Kleij reported on the photocatalytic synthesis of aryl‐substituted 6M‐CCs from γ‐substituted allyl carbonates, which, in turn, can be prepared from allyl alcohols.^[^ ^60^ ^]^ Using [Ru(bpy)_3_]Cl_2_ as the photocatalyst, several 6M‐CCs bearing different aromatic substituents were obtained with isolated yields ranging from 22% to 85% (**Scheme** **9**a). The proposed reaction pathway involves the formation of benzylic radical and cationic intermediates, and their stability likely influences the reaction outcome. In addition, the ROP of these new carbonates was studied using 1,5,7‐triazabicyclo[4.4.0]dec‐5‐ene (TBD) as the organocatalyst and benzyl alcohol as the initiator (Scheme 9b). Starting from disubstituted monomers, three different APCs were obtained with 2.6 ≤ *M*_n_ ≤ 5.2, and 1.23 ≤ *Đ* ≤ 1.42. Compared to PTMC, these new APCs exhibit relatively high *T*_g_ values, namely, 37, 56, and 65 °C, respectively for phenyl, naphthyl, and biphenyl substituents. Differently, polymerization attempts using three‐substituted monomers with quaternary carbon centers only result in the hydrolysis of the starting carbonates. Such difference was ascribed to steric hindrance around the carbonate, as evidenced by X‐ray analysis and further demonstrates the influence of the nature and substitution degree of the 6MCC on reactivity.

Lately, Kleij and co‐workers reported a new catalytic approach for the direct formation of functional bicyclic 6‐MCCs starting from β‐hydroxy‐cyclic epoxides and CO_2_.^[^ ^61^ ^]^

**Scheme 10:**
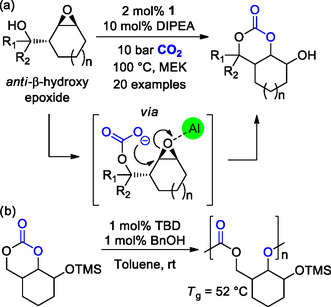
a) Direct synthesis of bicyclic 6M‐CCs from β‐hydroxy‐epoxides and b) following organocatalytic ROP.

In the presence of Al(III)‐complex **1** (see Scheme 6) and a selected base, namely, *N*,*N*‐diisopropylethylamine (DIPEA), the alcohol activates CO_2_ yielding a hemicarbonate intermediate that further evolves by ring‐opening the epoxide‐ring yielding the desired bicyclic β‐hydroxy‐6‐MCC (**Scheme** **10**a). It is important to note that the stereochemistry of the starting epoxide affects the reaction outcome: 6M‐CCs were obtained starting from the anti‐isomers, while the syn‐isomers only yielded the corresponding 5M‐CCs. The alcohol moiety of a selected carbonate was protected by reaction with TMSCl, allowing for organocatalytic ROP studies to obtain a new APC with *M*_n_ ranging from 5.5 to 8.6 kDa, *Đ* values from 1.20 to 1.34, and with a *T*_g_ of 52 °C (Scheme 10b).

**Scheme 11:**
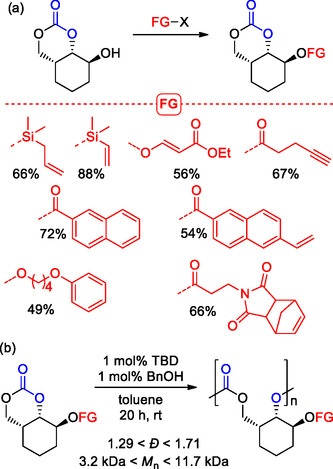
a) Synthesis of bicyclic 6M‐CCs and b) their ROP toward functional APCs with postmodification potential. Isolated yields are presented for each 6M‐CC product.

Later on, the same research group extended this new procedure for the synthesis of various bicyclic 6M‐CCs bearing different functional groups (**Scheme** **11**).^[^ ^62^ ^]^ ROP of such carbonates promoted by TBD was performed obtaining a series of different new functional APCs with *M*_n_ ranging from 3.2 to 11.7 kDa, and *Đ* values from 1.29 to 1.64. Notably, relatively high glass transition temperatures were recorded; in particular, for naphthoyl‐substitued APC a *T*_g_ of 106 °C was observed which is comparable to that of PCHC.

**Scheme 12:**
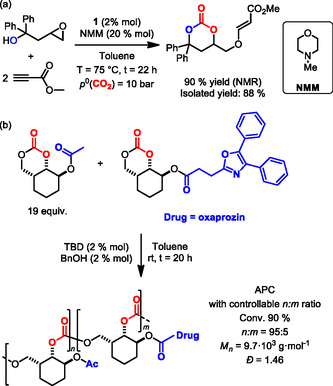
a) Catalytic domino three‐component synthesis of functional 6‐MCCs and b) synthesis of drug‐functionalized APC.

More recently, the same group expanded the scope of synthetically accessible 6M‐CCs monomers, reporting a modular three component domino approach combining an initial carboxylative cyclization of terminal epoxides derived β‐epoxy alcohols with an oxa‐Michael reaction step (**Scheme** **12**a).^[^ ^63^ ^]^ Using different electrophile coreactants the reaction was extended to 32 examples, with isolated yields ranging from 34% to 93%. This latter approach was further exploited for the synthesis of drug‐modified 6M‐CCs and their organocatalyzed ROP, accessing novel molecular and polymeric scaffolds through carbonate‐drug conjugation (Scheme 12b).^[^ ^64^ ^]^

## 3 Diols as a Direct Source of CCs

A valid and promising alternative to the cycloaddition of CO_2_ to epoxides to produce cyclic carbonates is the direct carbonylation of diols. The approach can be considered green for two reasons: the reaction leads only to water as a by‐product, and diols are starting materials that can be manufactured from biomass. For example, regarding the latter, a variety of diols or more in general polyols can be obtained from the hydrogenolysis of glycerol, sorbitol, etc., produced from biomass such as hemicellulose or cellulose.^[^ ^65^ ^]^ Since the carbonylation of diols with water as a by‐product is kinetically and thermodynamically disfavored, both homogeneous^[^ ^66^ ^]^ and heterogeneous catalysts^[^ ^67^ ^]^ have been developed together with the application of stoichiometric dehydrating process using, e.g., zeolites, nitriles, etc.^[^ ^68^ ^]^ However, this catalytic approach suffers from severe limitations. In addition to the limited catalyst stability at high CO_2_ pressure (>100 bar) and to reduced yield of reactions for the presence of an equilibrium with water,^[^ ^69^ ^]^ the synthesis of 6M‐CCs results particularly difficult.

In 2014, Tomishige et al. proposed a cascade catalyst based on CeO_2_ with 2‐cyanopyridine in which CeO_2_ simultaneously catalyzed the carbonylation of diols and hydration of 2‐cyanopyridine by water produced as a by‐product.^[^ ^70^ ^]^

**Figure 1:**
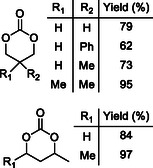
6M‐CCs obtained by CeO_2_‐catalyzed reaction of diols with CO_2_.

**Scheme 13:**
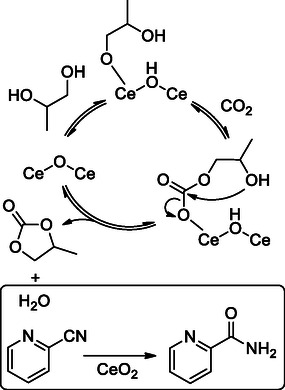
Ceria‐catalyzed CC synthesis from diols and CO_2_ using 2‐cyanopyridine as dehydrating agent.

They found that this catalytic system was efficient for the direct synthesis of various cyclic carbonates and reported, for the first time, high yield (up to 99%) and selectivity (from 77% to 99%) for a series of 6‐membered ring carbonates (**Figure** **1**). The proposed mechanism of action involves the adsorption of one of the —OH groups of a diol to the acidic sites of CeO_2_ to give cerium alkoxide, followed by the insertion of CO_2_ into the Ce—O bond that led to the alkyl carbonate species (**Scheme** **13**).

The nucleophilic attack of the second —OH diol group to the carbonyl carbon produces the cyclic carbonate with the release of H_2_O and the regeneration of CeO_2_. The removal of H_2_O from the equilibrium by hydration of 2‐cyanopyridine in the presence of ceria allows for high yields. However, this approach still requires high CO_2_ pressure (5.0 MPa) and temperatures (403–443 K).

**Scheme 14:**
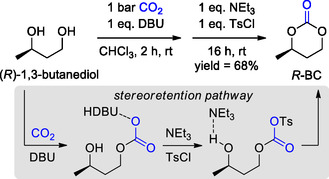
DBU‐mediated direct CC synthesis from CO_2_ and diols.

In this respect, 1 year later, Buchard et al. proposed an alternative method specifically for the synthesis of 6‐MCCs from 1,3 diols. The reaction uses only 1 atm of CO_2_ and proceeds at room temperature. Inspired by the work of Jessop, which reported the reversible carbonation of alcohols with CO_2_ in the presence of 1,8‐diazabicyclo[5.4.0]‐undec‐7‐ene (DBU), whose conversion was dependent on the reaction solvent and was almost complete in CDCl_3_,^[^ ^71^ ^]^ Buchard investigated the selective monoinsertion of CO_2_ into one alcohol moiety of 1,3‐diols in various deuterated solvents.^[^ ^72^ ^]^ The best results were obtained once again in chloroform. Despite the use of a nondesirable solvent, the addition of 1 equivalent of tosyl chloride and triethylamine after the DBU‐mediated monoinsertion of CO_2_ led to the rapid formation of the cyclic carbonate, overcoming the kinetic and thermodynamic limit of the cyclization (**Scheme** **14**).

The reaction mechanism may proceed either via an addition/elimination or an S_N_2 pathway, with retention or inversion of stereochemistry, respectively. Experiments with optically active diols result in retention of the initial stereochemistry and the authors proposed an addition/elimination mechanism, supported by density functional theory (DFT) calculations.

A greener method for the synthesis of 6‐MCCs, which overcame the use of chloroform as solvent, is the ring‐closing depolymerization proposed by Olsen.^[^ ^73^ ^]^ In the 1930s, Carothers et al. observed the reversible nature of the 6‐membered carbonates, thus opening the route to the application of this method.^[^ ^74^ ^]^

**Scheme 15:**
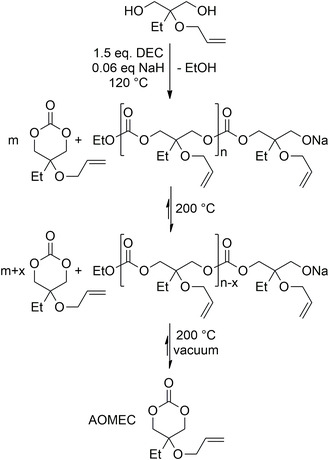
Oligomerization–depolymerization method for the synthesis of 2‐allyloxymethyl‐2‐ethyltrimethylene carbonate (AOMEC).

Interestingly, Olsen applied the depolymerization for the synthesis of functional 6M‐CCs, such as 2‐allyloxymethyl‐2‐ethyltrimethylene carbonate (AOMEC) (**Scheme** **15**). Starting from a trimethylolpropane allyl ether, an alloxyl‐functionalized 1,3‐diol, using diethyl carbonate (DEC) as condensation reactant and sodium hydride as an activator, the authors observed the formation of high molecular weight species. The depolymerization was performed directly after the condensation step in the same reaction vessel; due to the presence of an anionic environment the depolymerization reaction occurred without the aid of an additional ring‐closing depolymerization catalyst to form the cyclic carbonate.^[^ ^75^ ^]^

**Scheme 16:**
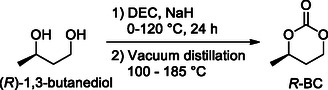
Synthesis of enantiopure (*R*)‐1,3‐butylene carbonate (*R*‐BC) from bio‐butylene glycol.

The preparation of enantiomerically pure cyclic carbonate starting from enantiopure of diols was carried out for the first time by Tsutsumi and Yasuda.^[^ ^76^ ^]^ They obtained the pure (*R*)‐ and (*S*)‐1,3‐butylene carbonate (*R*‐ and *S*‐BC) by reacting the corresponding enantiomerically pure 1,3‐butanediol with ethyl chloroformate in dilute tetrahydrofuran. Buchard prepared the *R*‐BC from bio‐butanediol (or bio‐butylene glycol, bio‐BG) using the synthetic approach reported above (Scheme 14),^[^ ^72^ ^]^ while Hillmyer, still starting from bio‐BG, adopted the greener method developed by Olsen. In the latter case, the *R*‐BC was easily recovered by distillation from the reaction mixture as reported in **Scheme** **16**.

Among the catalytic approaches reported in the literature for the synthesis of 6‐MCCs, of some relevance is the transesterification of diols catalyzed by methylcarbonate methyltrioctylphosphonium salt ([P_8881_][MeOCO_2_]).^[^ ^77^ ^]^

The use of 1,3‐butanediol and 2‐methyl‐1,3‐propanediol allowed the formation of the 6‐membered cyclic carbonate in very low yield with respect to other transesterification products while using 2‐methyl‐2,4‐pentandiol the cyclic carbonate was highly favored: indeed after 12 h, it was the sole reaction product in 96% yield (**Table** **2**).

**Table 2:** Transesterification of dimethyl carbonate with diols catalyzed by methyltrioctylphosphonium carbonate ([P_8881_][MeOCO_2_]).

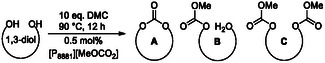				
1,3‐diol	Conv. [%]a)	**A** [%]	**B** [%]	**C** [%]
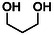	>99	<1	11	89
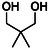	>99	1	18	81
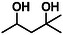	>99	96	3	–

a) Determined by ^1^H NMR spectroscopy.

Six‐membered ring carbonates work quite well as monomers in ROP, hence their synthesis is generally aimed at obtaining monomers for the production of novel polycarbonates, copolymers, or nonisocyanate polyurethanes. However, one of the limits in the use of such molecules as monomers is their scarce variety of functional groups that, if present, would confer targeted properties to the final material.

To this end, 2,2‐bis‐(hydroxymethyl)propionic acid (bis‐MPA) was considered a valuable monomer precursor due to the feasibility of introducing various functional groups via esterification. However, the synthesis of cyclic bis‐MPA‐carbonate monomer requires the use of toxic reagents, such as triphosgene, and also protection/deprotection leading to multistep procedures.

**Scheme 17:**
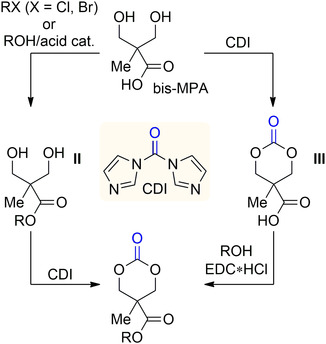
Synthesis of bis‐BPA‐based functional 6M‐CC using *N*,*N*′‐carbonyldiimidazole (CDI).

In 2021, Park and collaborators used bis‐MPA to develop a two‐step synthesis for a series of cyclic 6‐membered‐functionalized carbonates.^[^ ^78^ ^]^ Two potential strategies were investigated depending on the availability of the precursor: an alkyl (or benzyl/allyl) halide or an alcohol. More particularly, one strategy relies on the alkylation (or Fischer esterification) of the bis‐MPA carboxylic acid to afford the adduct **II**, followed by cyclization in acidic environment (**Scheme** **17**, left). The second strategy involved the selective cyclization of bis‐MPA to the corresponding carbonate carboxylic acid **III**, followed by esterification using EDC·HCl (Scheme 17, right). The critical cyclization step was overcome by using *N*,*N*′‐carbonyldiimidazole (CDI) as a carbonyl source.

**Scheme 18:**
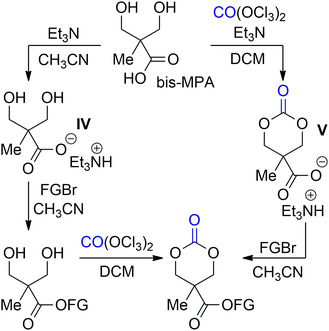
Synthesis of bis‐BPA‐based functional 6M‐CC via carboxylate intermediates.

Later on, Fukushima demonstrated that the organic carboxylate salt of the bis‐MPA cyclic carbonate (**IV**) was suitable for the formation of functionalized cyclic carbonate via esterification with alkyl bromides (**Scheme** **18**).^[^ ^50^ ^]^

The reaction proceeded with an S_N_2 mechanism in the presence of alkyl bromides with α‐carbonyl, allyl, and benzyl groups. The authors also explored the use of the intermediate **V** that enabled the use of higher temperatures for the esterification and to achieve higher conversions. Overall, the synthetic approach allowed for the attachment of diverse functional groups (FG) using commercially available modified α‐carbonylalkyl and benzyl bromides.

## 4 ROP of Larger CCs

Catalytic ROP represents an important process to afford polycarbonates. However, while the ROP of 6M‐CCs is well documented, the polymerization of large cyclic carbonates remains challenging due to their low ring strain and entropy‐driven mechanism. Within this realm, biocatalysis plays a crucial role, especially in the synthesis of APCs. Thus, biocatalytic ROP was introduced by Kobayashi and involves the use of lipases as catalysts to promote the polymerization of cyclic monomers.^[^ ^79^ ^]^

The lipase B from *Candida antarctica* (CALB) is commonly employed in these reactions due to its high catalytic activity and stability. One of the key advantages of lipase‐catalyzed ROP is its environmental friendliness if compared with metal‐mediated catalysis. Furthermore, the use of lipase in ROP allows for a high degree of control over the polymerization process, by adjusting the reaction conditions to obtain the target molecular weight and polydispersity of the resulting polymer, enabling the production of materials with tailored properties. This level of control is particularly valuable in the development of advanced materials for applications in areas such as biomedicine and electronics.^[^ ^80^ ^]^

**Scheme 19:**
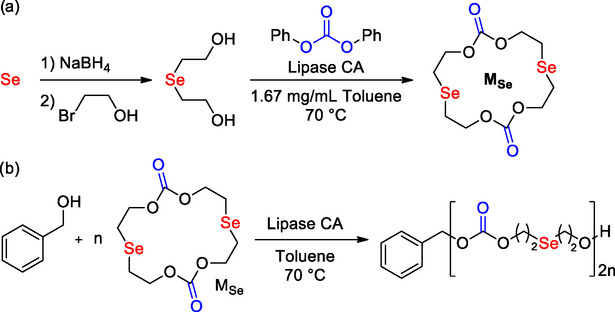
a) Biocatalytic synthesis of selenium containing cyclic dicarbonate *M*_Se_ and b) its ROP promoted by lipase CA.

In 2018, Lang and co‐workers reported the preparation of selenium‐containing aliphatic polycarbonates via ring‐opening polymerization of the large cyclic dicarbonate monomer promoted by lipase.^[80b]^ In this work cyclic diethylene selenide carbonate dimer (*M*_Se_) was prepared using an efficient two‐step procedure (**Scheme** **19**a) that involves the reaction of selenium powder with sodium borohydride, and bromoethanol to give di(1‐hydroxyethylene)selenide, followed by an intermolecular cyclization with diphenyl carbonate using lipase CALB as catalyst (Scheme 19a). The ROP of the resulting *M*_Se_ was then investigated by employing the same lipase (CALB) and benzyl alcohol as initiator in toluene at 70 °C, due to the high melting temperature of the *M*_Se_ (Scheme 19b). GPC analysis showed the quasi‐narrow molecular weight distributions, in line with other enzymatic systems but slightly wider than analogue materials prepared via metal or organic catalysis.^[^ ^81^ ^]^

The authors also investigated the ROP kinetics; fast monomer conversion was observed in 4 h with [BnOH]_0_/[*M*_Se_]_0_ = 1/20 mol/mol, and ln([*M*]_0_/[*M*_t_]) versus reaction time showed first‐order dependent kinetics that support the living polymerization characteristic.

**Scheme 20:**
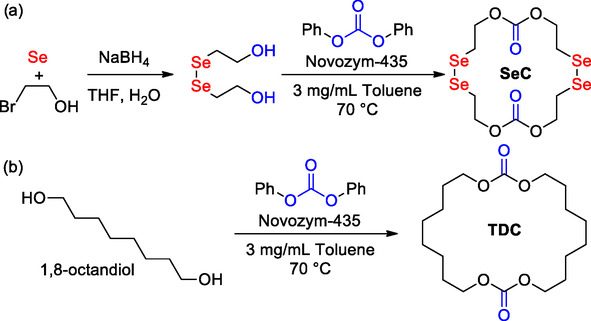
a) Biocatalytic synthesis of diselenium containing cyclic dicarbonate SeC and b) cyclic dicarbonate TDC.

**Scheme 21:**
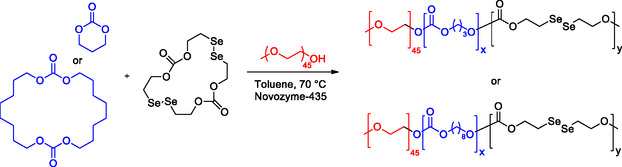
Copolymerization of SeC with TMC (control) and TDC.

Recently, Zhang disclosed a comprehensive study on the synthesis and copolymerization of similar selenium‐containing cyclic carbonates by enzyme‐catalyzed ROP, with the primary goal of creating biodegradable polycarbonates with unique properties.^[^ ^82^ ^]^ The focus was on the copolymerization of the diselenium‐containing cyclic dicarbonate SeC, with TMC and the large cyclic dicarbonate 1,3,12,14‐tetraoxacyclodocosane‐2,13‐dione (TDC) (**Scheme** **20**). SeC and TDC were synthesized in using enzymatic ring‐closure in 30% and 60% yield, respectively. The successful synthesis of these compounds was confirmed through NMR spectroscopy and mass spectrometry. Then, homopolymerization and copolymerization of these compounds were investigated, employing Novozyme‐435, a supported form of CALB, as the catalyst and mPEG_45_ as the initiator at 70 °C. It was found that the homopolymerization of TDC was more challenging compared to TMC, which was employed as control. Furthermore, copolymerization was carried out with SeC and either TMC or TDC, showing better results with TMC (mPEG_45_‐TMC‐SeC; 1:60:5; conv. 98%) than TDC (mPEG_45_‐TDC‐SeC; 1:10:5; conv. 78%) (**Scheme** **21**).

Thermal properties of the copolymers were studied as well. The copolymers exhibited a two‐stage decomposition process at 250 and 380 °C, respectively. The obtained amphiphilic block copolymers exhibit the capability to self‐assemble into nanoparticles. Thus, the authors studied their responsiveness to reactive oxygen species (ROS) since the Se—Se bond is well known to undergo cleavage into Se=O/Se—OH in the presence of peroxides. Over production of ROS leads to oxidative stress related to several diseases (e.g., cancer and atherosclerosis) and may serve as trigger for responsive drug‐delivery systems.^[^ ^83^ ^]^ Interestingly, the mPEG‐b‐(PTDC*‐co*‐PSeC) copolymers demonstrated ROS responsiveness, and upon oxidation with H_2_O_2_, the micelles disassembled indicating potential for further applications.

Lipase‐catalyzed ROP represents a breakthrough in the field of polymer chemistry. Its ability to produce high‐quality, sustainable polycarbonates has made it a valuable tool in the development of new materials from a sustainable perspective.

**Scheme 22:**
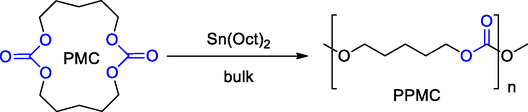
Synthesis of poly(pentamethylene carbonate) (PPMC) via ROP promoted by Sn(Oct)_2_.

As research in this area continues, the scope and impact of lipase‐catalyzed ROP are expected to continue to grow. On the other hand, metal‐catalyzed ROP of large cyclic carbonates, although still an important strategy, is scarce in recent literature. In fact, to our knowledge, one of the last works reported was in 2010 by Feng and co‐workers.^[^ ^84^ ^]^ The authors reported a detailed study on the synthesis of poly(pentamethylene carbonate) (PPMC) via ROP of the corresponding pentamethylene carbonate (PMC) catalyzed by Sn(Oct)_2_. The authors successfully synthesized PPMC with high molecular weights (up to 1.7 × 10^5^ g mol^−1^) demonstrating the efficiency of Sn(Oct)_2_ as a catalyst (**Scheme** **22**).

Thermal properties of the polymers were studied using differential scanning calorimetry and the results provided valuable insights into the structure–property relationship of the polymers. The authors discussed the influence of the number of methylene groups per repeat unit (Num_c_) on the crystallization rate and melting temperature (*T*_m_) of the polymers. However, this so‐called odd–even effect is related to the number of CH_2_ groups and represents an important feature for regulating the performance and expanding the applications of APCs as already reported by Matsumura.^[^ ^85^ ^]^

In 2023, Guo et al. reported a study on the alkali cation assisted ROP of large cyclic carbonates.^[^ ^86^ ^]^ The study explores the development of engineering polymers with controllable physical properties through ROP promoted by alkali metals of macrocyclic carbonates (MCs), in terms of thermodynamic and kinetic factors. The research investigates the use of a binary catalytic system combining TBD with alkali cations (Li^+^, Na^+^, K^+^) to modulate the polymerization process with the aim of understanding how these catalysts affect the polymerization process. Tetraethylene glycol carbonate (4EGMC) was selected as benchmark substrate and its polymerization proceed smoothly using TBD/Na^+^ system, achieving high conversion rate (94%) within 30 min, resulting in poly(tetraethylene glycol carbonate) (P4EGMC) with a molecular weight (*M*_n_) of 5.9 kg mol^−1^ and a narrow dispersity (*Đ* = 1.15).

**Scheme 23:**
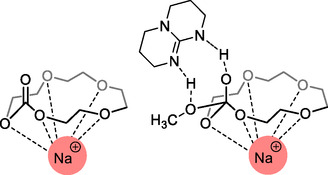
Tetraethylene glycol carbonate (4EGMC)/Na^+^ structure (left), and rate‐determining step of ROP activated by TBD (right).

Furthermore, the study of thermodynamics, backed by both experimental data and DFT, unveils that sodium cation forms a coordination complex with MC, resulting in conformationally restricted ring configurations (**Scheme** **23**). This alters the thermodynamic driving force of polymerization, shifting from entropy‐driven to an enthalpy‐driven process. Furthermore, the reaction mechanism supported by DFT shows that the sodium cation plays a pivotal role as a catalyst in the polymerization process due to the coordination of the sodium ion (Na^+^) with MC and the initiator. Finally, the authors investigated the role of cation comparing polymerization kinetics of TBD catalyzed reactions in the presence of Li^+^, Na^+^, and K^+^. As expected, changing the cation affects the polymerization kinetic with observed polymerization rate following the order Li^+^ > K^+^ > Na^+^. This difference was ascribed to the competing binding of cations to the polymer chain, which interfere with the proposed monomer activation mechanism.

Organocatalysis has emerged as a potent substitute for conventional metal‐based catalysts and biocatalysis in ROP. Advances in this field have reached a stage at which not only they are superior, cost‐effective, and user‐friendly organocatalysts available for routine polymerizations, but they also have the capability to regulate polymer structures accurately. Additionally, it is now possible to polymerise ROP monomers that were previously extremely difficult to polymerize using other methods.^[^ ^87^ ^]^

Recently, Lang et al. explored the ROP of MCs using TBD as the organocatalyst to prepare copolycarbonates focusing on their properties.^[^ ^88^ ^]^ The study successfully demonstrated the use of TBD as an efficient catalyst for the ROP of MCs, achieving high monomer conversions affording block co(polycarbonates), consisting of two or more homopolymer subunits linked to each other, with modular properties. The results showed that temperature plays a crucial role in the polymerization process, with high conversion observed at 70 °C in toluene and significantly lower at room temperature.

**Scheme 24:**
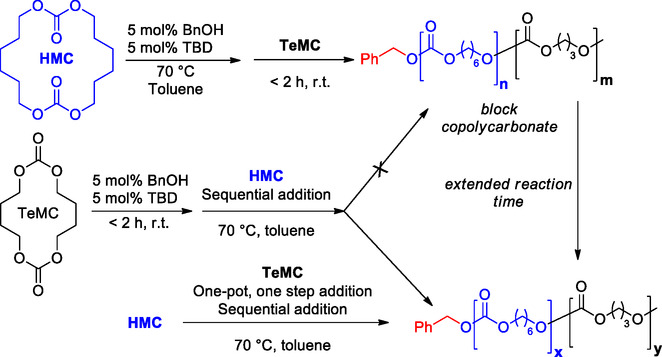
Approaches to block polymerization and effect of extended reaction time.

In terms of yield and polydispersity, the polymerization of hexamethylene carbonate (HMC) at 70 °C achieved a high conversion of 97% within 8 h, resulting in homopolycarbonates with a narrow molecular weight distribution. Similarly, high monomer conversions (>98%) were obtained for PMC and heptamethylene carbonate (HeMC) monomers at 70 °C, whereas the ROP of tetramethylene carbonate (TeMC) showed a slower polymerization rate due to its poor solubility in toluene. The study also demonstrated the ability to control polymer topology by adjusting the polymerization time, with longer times leading to a gradual transformation from block to random copolymers due to transesterification (**Scheme** **24**).

**Scheme 25:**
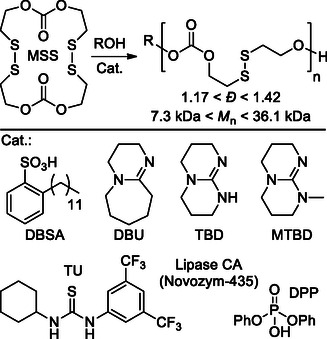
ROP of disulfide‐functional macrocyclic carbonates (MSS) and the organocatalysts tested.

The same authors published a follow‐up of this work in the same year.^[^ ^89^ ^]^ In this latter study, they explored several organocatalysts for the efficient and controlled ROP of disulfide‐functional macrocyclic carbonates (MSS), offering an alternative to traditional enzymatic catalysis. Even in this case, TBD showed the best results when the polymerization was conducted at room temperature in dichloromethane with benzyl alcohol (BnOH) as the initiator. The results demonstrated that TBD could achieve a monomer conversion of 95% within 2 h, significantly higher than other catalysts like DBU, which only reached 80% conversion after 8 h. This high efficiency is attributed to the dual activation mechanism of TBD, which activates both the initiator and the monomer through hydrogen bonding (**Scheme** **25**).^[^ ^90^ ^]^ The molecular weights of the resulting polymers were close to the theoretical values, with narrow molecular weight distributions (*Đ* = 1.17–1.28), indicating a controlled polymerization process. The study also compared the organocatalytic method with enzymatic catalysis using lipase CA (Novozyme‐435). The enzyme‐catalyzed ROP required higher temperatures (70 °C) and longer reaction times (12 h) to achieve similar monomer conversion rates, with broader molecular weight distributions (*Đ* = 1.38–1.45).

In terms of the synthesis of starting MCs and their application in ROP, more recently Odelius and co‐workers disclosed a novel approach to synthesize them in high yields.^[^ ^91^ ^]^

**Scheme 26:**
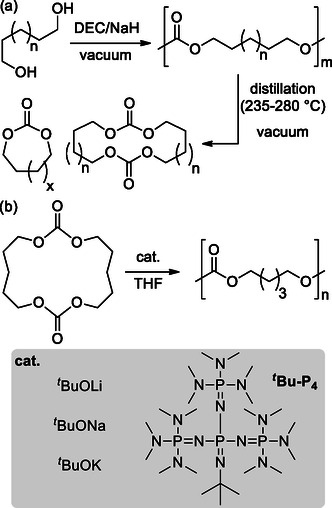
Synthesis of aliphatic MCs and their application in ROP reported by Odelius.

The optimized conditions for the synthesis of MCs resulted in yields ranging from 62% to 80% employing different diols as substrates; sodium hydride (NaH) was used for the first step that consist of the polycondensation of diols and diethyl carbonate (**Scheme** **26**a). The concentration of NaH was critical, with the highest yield obtained at the lowest loading (0.004 eq.). The depolymerization process also showed high selectivity and yields, with isolated yields ranging from 70% to 85% for different MCs (Scheme 26a). The authors also explored the use of organic catalysts and anionic initiators for the ROP of MCs, with ^*t*^Bu‐P_4_ and sodium *tert*‐butoxide (^*t*^BuONa) showing high conversion rates (>97%) and fast polymerization (less than 10 s) at ambient temperature (Scheme 26b). The study also highlighted the “odd–even” effect, where the polymerization rate depended on the number of CH_2_ units between carbonate linkages, influencing even the structural conformation of the resulting products. In addition, single crystal X‐ray diffraction analyses revealed that MCs with odd numbers of CH_2_ groups had a *syn* conformation, leading to higher polymerization rates compared to their even‐numbered counterparts.

Similarly, Li, Huang and co‐workers developed a “ring‐to‐ring” closed loop process for the preparation of large cycle organic carbonates and dicarbonates in high purity (93−99 mol%) and yield (90−95%), upon *N*‐heterocyclic carbene‐mediated depolymerization of cyclic aliphatic polycarbonates. Interestingly, the reversible polymerization/depolymerization approach allowed also for the preparation of a library of cyclic aliphatic polycarbonates, consisting of long (CH_2_)_*n*_ segments (5 ≥ *n* ≥ 8), with high molecular weight (111.4−147.9 kg mol^−1^, DP = 468−568), obtained with high monomers’ conversion (94−96 mol%), in mild experimental conditions (r.t.). As previously observed, the resulting cyclic APCs displayed thermal stabilities significantly higher compared to their linear analogues.^[^ ^92^ ^]^

## 5 Circular Polymers and Closed‐Loop Recycling

**Figure 2:**
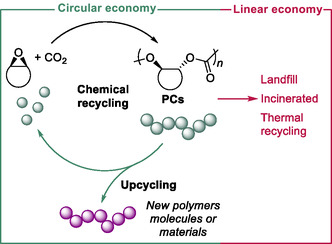
Linear versus circular economy (polycarbonate chemical recycling to monomers or polymer upcycling).

Designing new types of polymers with the end‐of‐life in mind, in which closed‐loop recycling is considered within the monomer and polymer design, is now one of the main research challenges.^[^ ^93^ ^]^ Currently, commodities plastics at their end‐of‐life are either valorized through primary (closed‐loop) and secondary (cascade) recycling or, more commonly, combusted for energy production.^[^ ^94^ ^]^ However, most plastics are relegated to landfill or are leaked into the environment. Most of today's polymers are in fact designed on a linear economy that does not address the material end‐of‐life issues thereby increasing concerns about feedstock availability and enhancing the current global plastics pollution crisis (**Figure** **2**).^[^ ^95^ ^]^ Differently, to address plastic problems, both related to the sourcing or feedstocks and the end‐of‐life of these materials, circular polymer design can represent a viable solution. Circular polymers are defined as inherently, selectively, and expediently depolymerisable to their monomer state once their kinetic barriers of deconstruction are overcome.

This enables not only the ideal shortest chemical circularity but also tuneable performance properties. In an ideal closed‐loop process, the same catalytic system can be used for both polymer synthesis and for their recycling. A priori, any polymer could be made reversible by tuning the thermodynamic equilibrium of polymerization and depolymerization processes by adjusting reaction conditions, such as temperature, concentration, pressure, the presence of catalyst, etc. However, in reality, energy barriers play a fundamental role in determining whether a polymer can be practically and efficiently depolymerized to its monomer (chemical recycling to monomers, CRM) and then reobtained from such monomer to complete the polymer‐monomer‐polymer life cycle. This is determined by the kinetics of the forward and backward reactions in terms of rate, energy input, and selectivity and a thermodynamic measure of polymerisability (from the monomer's perspective) or depolymerisability (from the polymer's perspective) can be quantified by ceiling temperature (*T*_c_).^[^ ^96^ ^]^ The *T*_c_ determines the relative stability of monomer–polymer states at different working temperature regimes and it is defined as the temperature at which the de/polymerization reactions reach an equilibrium state.^[^ ^96^ ^]^ To accelerate the depolymerization rate and thus reach the equilibrium in an operable time, the use of a highly effective catalyst could significantly lower the energy barrier.

**Scheme 27:**
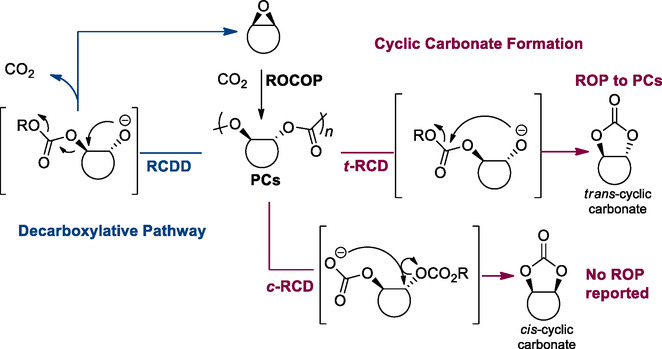
Proposed polycarbonates depolymerization pathways. Ring‐closing decarboxylative depolymerization pathway to epoxides (RCDD, left), and cyclic ring‐closing depolymerization pathway to cyclic carbonate (RCD, right).

While many examples of the design of monomers for closed‐loop systems have been reported for polyester‐based structures, polycarbonates have been less explored. Ahead of getting into the most relevant current examples, to gain more insight into the design processes of circular polymers, the two main depolymerization mechanisms need to be taken into consideration: the first one leads to the formation of cyclic carbonates, while the second decarboxylative pathway leads to the formation of epoxy monomers.^[^ ^96^ ^]^ The ring‐opening copolymerization of an alicyclic epoxide and CO_2_ generates a polycarbonate with *trans‐*backbone stereochemistry. The decarboxylative pathway occurs through an alkoxide chain‐end backbiting mechanism, forming a *cis* alicyclic epoxide and CO_2_ by attack on the proximate backbone ring (RCDD in **Scheme** **27**). Differently, a *trans*‐alicyclic cyclic carbonate can be formed through a second possible backbiting reaction from the same chain end that follows a *trans*‐ring‐closing depolymerization (*t*‐RCD), with formation of a *trans*‐alicyclic carbonate by alkoxide attack on the closest carbonyl (*t*‐RCD). Alternatively, a carbonate chain‐end attack at the vicinal position generates a *cis* alicyclic carbonate through *cis*‐ring‐closing depolymerization (*c*‐RCD). However, as mentioned above, *cis*‐5‐membered cyclic carbonates do not undergo ring‐opening polymerization, therefore the *c*‐RCD pathway is considered a reactive dead. Ideally, in a depolymerization process, the reaction conditions are adjusted to reach the full polymer deconstruction toward the constituent monomers and limit the formation of oligomers. The depolymerization and therefore the chemical recycling of polycarbonates gained increasing attention in the last decades. For previous examples, we refer to exhaustive reviews published on this topic,^[^ ^96^ ^]^ while in the present review, we will focus on works published lately.

**Scheme 28:**
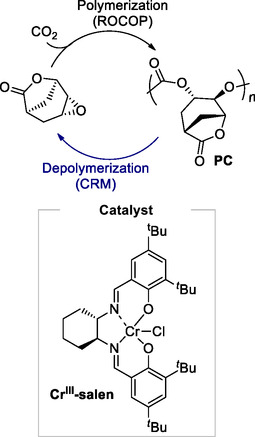
Circular polymer design and closed‐loop recycling of polycarbonates via decarboxylative mechanism promoted by Cr(III)‐salen catalyst.

A recent work was reported by Chen and co‐workers in which a multiple‐materials approach through the design of a bifunctional monomer was discussed.^[^ ^97^ ^]^ The bifunctional monomer can not only orthogonally polymerize into two different types of polymers, specifically lactone‐based polyester and CO_2_‐based polycarbonate, but the resultant polymers and their mixture can also be depolymerized back to the single, original monomer in the presence of a phosphazene catalyst. Furthermore, the epoxide‐polycarbonate closed‐loop circularity was explored using a Cr(III)‐salen catalyst (**Scheme** **28**) to promote the ROCOP between the epoxy‐monomer and CO_2_ and later, to selectively depolymerise the polycarbonate to its epoxy monomer in the presence of PPNN_3_ [bis(triphenylphosphine)iminium azide] through a decarboxylative pathway. Depolymerization was conducted at 160 °C, achieving a selectivity of epoxide formation >99% and a yield of 69% in 5 min.

**Scheme 29:**
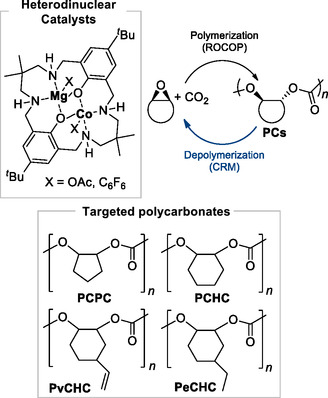
Closed‐loop polycarbonate recycling using a heteronuclear Mg(II)Co(II) catalyst system.

Williams and co‐workers have instead developed a heterodinuclear Mg(II)Co(II) catalyst that promotes polycarbonate chemical recycling to epoxides and CO_2_ that occurs through a chain‐end depolymerization mechanism (**Scheme** **29**). The protocol was extended to poly(cyclohexene carbonate) (PCHC), for the preparation of epoxides and carbon dioxide using solid‐state conditions, in contrast to many other CRM strategies that rely on high dilution. The depolymerizations gave very high activity and selectivity (monomer selectivity >99%, 0.02 mol% catalyst loading, *T* = 140 °C). Reactions were also performed in the presence of air, without impacting the rate or selectivity of epoxide formation and could be scaled up to a 2 g scale to isolate the epoxides in up to 95% yield with >99% selectivity with catalyst recyclability tested over four catalytic cycles.

Later, the same group described the use of the organometallic Mg(II)Co(II) catalyst to develop a closed‐loop protocol. The binuclear catalyst combined high productivity, stability under low catalyst loading, and the highest polymerization control to yield polycarbonates with monomodal, narrow molecular weights distributions and *M*_n_ values ranging from 4 to 130 kg mol^−1^.^[^ ^98^ ^]^ All these high molecular weight polymers are fully recyclable, either by reprocessing or by using the Mg(II)Co(II) catalyst for highly selective depolymerizations to epoxides and CO_2_. Among all the polycarbonates tested, poly(cyclopentene carbonate) showed the fastest depolymerization rates, achieving an activity of 2500 h^−1^ and >99% selectivity for cyclopentene oxide and CO_2_.

**Scheme 30:**
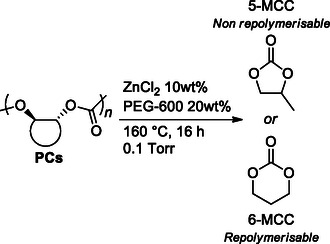
APC depolymerization by reactive distillation to 5‐membered and 6‐membered cyclic carbonates.

A ZnCl_2_/poly(ethylene glycol) (PEG) catalyst system was also reported for the depolymerization of polycarbonates derived from cyclic monomers at high temperatures (*T* = 160 °C) and under reduced pressure (**Scheme** **30**).^[^ ^99^ ^]^ Poly(propylene carbonate) and poly(trimethylcarbonate) could be depolymerized to give the corresponding 5‐membered cyclic carbonates (92% isolated yield) and 6‐membered cyclic carbonates (85% isolated yield), in excellent yields. The combination of ZnCl_2_ and PEG‐600 (10 and 20 wt%, respectively) under vacuum can readily and selectively depolymerize polyesters and polycarbonates with high ceiling temperatures (*T*_c_ *> *200 °C) back to their original monomers. Mechanistic experiments implicate a random chain scission mechanism and a catalyst structure containing one equivalent of ZnCl_2_ per ethylene glycol repeat unit in the poly(ethylene glycol).

**Scheme 31:**
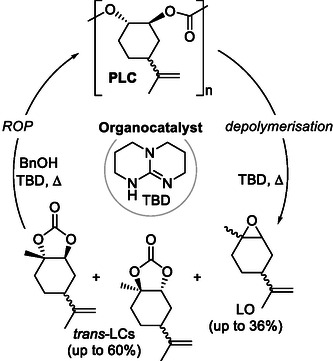
Organocatalytic bio‐derived PCs recycling to cyclic carbonates.

Moving from metal‐catalyzed protocols to an organocatalyzed protocol, the depolymerization of poly(limonene carbonate) (PLC) was performed providing access to its *trans*‐configured cyclic carbonate as the major product (**Scheme** **31**).^[^ ^100^ ^]^ The heterocyclic base TBD, in particular, offers a unique opportunity to break down polycarbonates via end‐group activation or main chain scission pathways as supported by various controls and computational analysis. *Trans*‐limonene carbonate (obtained with an isolated yield of 55%) could be converted back to its polycarbonate via ring‐opening polymerization using the same organocatalyst in the presence of an alcohol initiator (benzyl alcohol), thus offering a potential circular and practical route for polycarbonate recycling.

In analogy, the same research group has reported the selective depolymerization of fatty acid‐derived polycarbonates to the corresponding 5‐membered cyclic carbonate (isolated in 76% yield) promoted by TBD in toluene at *T* = 110 °C.^[^ ^101^ ^]^

To date, alternative depolymerization protocols have been reported in the presence of enzymes. The biocatalytic degradation and polymerization using an enzyme were carried out with respect to the establishment of a sustainable chemical recycling system for PTMC which is a potentially biodegradable synthetic plastic.^[^ ^102^ ^]^ The enzymatic transformation of PTMC using CALB in acetonitrile at 70 °C afforded the corresponding readily polymerizable cyclic monomer, trimethylene carbonate in a yield of up to 80%.

**Scheme 32:**
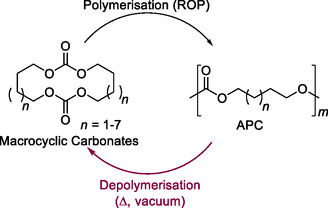
High vacuum selective depolymerization to cyclic dicarbonates.

Finally, polycarbonate chemical recycling to monomers has also been explored in the absence of a catalytic system using the favorable formation of cyclic dicarbonates (**Scheme** **32**). Depolymerizations were achieved with 0.05 mbar vacuum at high temperature (240−260 °C) achieving isolated yields ranging from 70% to 85% with high selectivity (95−99 mol%). These yields were slightly higher compared to the two‐step depolymerization system. To explain these results, the authors suggested that ROP leads to a more defined polymer structure with fewer side reactions, such as alcoholysis and dehydration, commonly seen during polycondensation.^[^ ^103^ ^]^

## 6 Conclusions

Large cyclic organic carbonates with ≥6 atoms within the heterocyclic rings gained increasing importance for the preparation of biocompatible, elastomeric APCs containing bridging units with more than two carbon atoms. This review provides an up‐to‐date survey of the reported methodologies for the synthesis of polymerizable cyclic organic carbonates (6M‐CCs and larger), with an emphasis on environmentally friendly and sustainable approaches. In particular, we highlight the use of diols (including bio‐derived ones) and nonhazardous carbonyl sources, such as linear organic carbonates, under mild reaction conditions. We emphasize the fact that 6M‐CCs have lower thermodynamic stability compared to their 5‐membered counterparts, making them ideal candidates for the production of APCs through ROP. This process can be facilitated by both organo‐ and biocatalysts to obtain well‐defined copolymers with tailored thermomechanical properties.

Interestingly, these polycarbonates can be easily obtained via ROP. The main factor limiting their accessibility is the restricted synthetic availability of large cyclic carbonates, ascribed to their limited thermodynamic stability. A promising and sustainable synthetic approach relies on the direct carbonylation of diols, allowing for the utilization of biomass‐derived diols, combined with formation of low molecular weight by‐products, e.g., alcohols and/or water. On one hand, the use of linear carbonates results in more favorable reaction conditions, but on the other it shifts the problem to the synthesis of such reagents. For example, DMC and DEC are formed by reaction of phosgene with methanol and ethanol, respectively.^[^ ^104^ ^]^ Consequently, the search for green and sustainable processes for the production of linear carbonates (e.g., by direct use of carbon dioxide) is an active research topic, which is strongly related to the production of sustainable polycarbonates.^[^ ^105^ ^]^ Moreover, the preparation of large cyclic carbonates still represents a synthetic challenge. Indeed, current procedures require very high dilution reaction conditions, the use aromatic and halogenated solvents, resulting in generally low isolated yields limiting the sustainability of the entire processes. For these reasons, further research is urgent to establish general, more efficient, and sustainable methodologies for the synthesis of large cyclic carbonates.

Additionally, recent advances in the controlled depolymerization of APCs are discussed, emphasizing the controlled chemical depolymerization strategies that selectively produce cyclic carbonate or epoxide monomers. Although both thermal and catalytic depolymerization routes are attracting in terms of circular economy, they suffer the limit of using high reaction temperatures. These high energy intensive processes increase the cost of the final recycled monomers/materials, rendering the overall processes uneconomical. This analysis highlights both the challenges and the opportunities in the synthesis and chemical recycling of APCs, pointing to their potential as circular, sustainable alternatives in the field of plastics.

In summary, we provide a comprehensive perspective on the state of APC synthesis, offering insights into the most promising and sustainable methods as well as the future direction for their chemical recycling and circular economy applications.

## Conflict of Interest

The authors declare no conflict of interest.
